# Radiofrequency Catheter Ablation: How to Manage and Prevent Collateral Damage?

**DOI:** 10.19102/icrm.2020.110901

**Published:** 2020-09-15

**Authors:** Mohammad El Baba, Dean Sabayon, Marwan M. Refaat

**Affiliations:** ^1^Electrophysiology Section, University Hospitals Cleveland Medical Center, Cleveland, OH, USA; ^2^Electrophysiology Section, Emory University Hospital, Atlanta, GA, USA; ^3^Department of Internal Medicine, Division of Cardiology/Electrophysiology Section, American University of Beirut Medical Center, Beirut, Lebanon; ^4^Department of Biochemistry and Molecular Genetics, American University of Beirut Faculty of Medicine, Beirut, Lebanon

**Keywords:** Ablation, atrial fibrillation, collateral damage, management, ventricular tachycardia

## Abstract

Radiofrequency catheter ablation has become the standard of care for the management of various arrhythmias and, in fact, the first-line therapy for many tachyarrhythmias. It entails creating scar tissue in the heart in regions where abnormal impulses form or propagate to restore normal cardiac conduction. As the heart is a complex organ and is surrounded by and related to many other anatomical structures, it is important to avoid the collateral damage that can happen from radiofrequency (RF) ablation on the endocardium as well as on the epicardium. This review explores methods for mitigating or limiting collateral damage during catheter ablation.

## Introduction

Radiofrequency (RF) catheter ablation has become the standard treatment for various arrhythmias and is the first-line therapy for treating many tachyarrhythmias.^1^ RF ablation adopts a high-frequency (500–750Hz) current to produce lesions through the process of thermal injury. Resistive heating damages the tissue in direct contact with the ablation catheter. Deeper tissues are heated and damaged by heat conduction.^2^ To cause tissue necrosis, the temperature at the electrode–tissue interface must be at least 50°C but, at temperatures approaching 100°C, a coagulum of denatured proteins forms on the catheter tip, limiting the delivery of current and increasing the risk of thromboembolic complications.^3^

As the heart is a complex organ and is surrounded by and related to many other structures, it is important to avoid any collateral damage linked to RF ablation on both the endocardium and the epicardium.

Complications vary depending on the procedure but serious complications are rare (eg, death, myocardial infarction, or stroke occurring in 0.05%–0.01%), with a higher risk of stroke correlated with atrial fibrillation (AF) ablation.^4^ Heart block requiring a permanent pacemaker is rare and occurs in 0.5% of catheter ablations. Vascular access complications occur in 2% to 4% of cases and cardiac tamponade occurs in 1% to 2% of cases. Phrenic nerve (PN) damage may be found especially after AF ablation. Also, with AF ablation, pulmonary vein (PV) stenosis may be observed. Meanwhile, atrioesophageal fistulae are extremely rare.

In this review, the complications and types of collateral damage from RF ablation of various arrhythmias as well as the measures to avoid them will be discussed.

### Prevention and management of complications and collateral damage in atrial fibrillation ablation

AF is the most common sustained arrhythmia, with significant morbidity and mortality rates. PV isolation has been reported to be an effective strategy for the treatment of symptomatic AF. However, despite developments in the technology and improvements in the techniques, catheter ablation for AF remains a highly complex procedure with a non-negligible risk of complications. Major complications have been reported to appear in up to 5.2% of procedures,^5–7^ while death occurs in one out of every 1,000 cases.^8^ In a systematic review by Cappato et al., the causes of deaths in 32,569 patients included tamponade in eight patients (including one case later than 30 days postprocedure), stroke in five patients (including two cases later than 30 days postprocedure), atrioesophageal fistula in five patients, and massive pneumonia in two patients. Myocardial infarction, intractable torsade de pointes, septicemia, sudden respiratory arrest, extrapericardial PV perforation, occlusion of both lateral PVs, hemothorax, and anaphylaxis were reported to be responsible for one death each, while asphyxia from tracheal compression secondary to subclavian hematoma, intracranial bleeding, acute respiratory distress syndrome, and esophageal perforation from an intraoperative transesophageal echocardiographic probe were causes of one late death each, respectively.^8^

#### Pulmonary vein stenosis.

The PVs have been demonstrated to be substantial in AF initiation and maintenance.^9,10^ Around 88.8% of ectopic beats initiating AF originate from the PVs. PV isolation is now performed in AF ablation procedures.

During the early work with AF ablation and before the improvement of clinical experience, isolation of the PVs was carried out by applying RF lesions within the veins or at the venoatrial junction. Thus, PV stenosis has been a major concern after AF ablation. Early reports showed an incidence of PV stenosis of between 8% and 15%.^11–13^ Factors contributing to the development of PV stenosis included the application of energy inside the veins and inappropriate energy delivery.^12,13^ It remains unclear exactly how PV stenosis develops. Responses to thermal injury within the PVs, which include intimal proliferation with organizing thrombus, necrotic myocardium in various stages of collagen replacement, endovascular contraction, and the proliferation of elastic lamina, could be a potential explanation.^14^

The most common symptom resulting from severe PV stenosis is dyspnea on exertion. Also, dyspnea at rest, recurrent cough, pleuritic chest pain, flu-like symptoms, and hemoptysis have been reported.^15^ Patients with symptomatic PV stenosis are often diagnosed late due to the nonspecific nature of their symptoms.^16^

The location and extent of the stenosis are best evaluated by computed tomography (CT). Lung perfusion scans also can be beneficial, especially to determine the effect of the stenosis. Symptoms of PV stenosis develop when the perfusion of the affected lobe falls below 20% or the perfusion of the entire lung on the affected side falls below 25%.^17^

PV angioplasty remains the best treatment option for symptomatic PV stenosis. In a study by Prieto et al., stent angioplasty resulted in less restenosis than dilation, particularly for stents measuring 10 mm or larger.^17^ Here, the restenosis rates were 72% for dilation versus 33% for stenting (p < 0.001) over a mean follow-up period of 25 months. Time to restenosis was greater for stent angioplasty (p = 0.003). Conversely, the management of asymptomatic patients is challenging and risks should be weighed with the potential benefits of the procedure.^12^

Overall, the incidence of severe PV stenosis is decreasing. In one report, the incidence rate of severe PV stenosis was less than 1% and the incidence rate of symptomatic PV stenosis necessitating intervention was negligible.^18^ This is mainly due to the change in the ablation technique by adopting an antral ablation approach.^19^ The wide diameter of the antrum prevents PV stenosis, even in the presence of scar retraction at the ablation sites. The adoption of this new ablation technique has drastically decreased symptomatic PV stenosis.^12^

However, some areas of the PV, such as the ridge that separates the left PVs from the left atrial (LA) appendage, make it impossible to conduct PV ablation from a distance. In addition, the esophagus sometimes lies in close proximity to the PVs on one side and ablation must be performed on the PV itself to avoid damaging the esophagus.

#### Atrioesophageal fistula.

Atrioesophageal fistula is as an extremely rare (0.04%) but catastrophic complication of the catheter ablation of AF.^20^

The esophagus lies posterior to the LA with a variable course relative to the LA adjacent to the right or left PVs or the posterior wall.^21,22^ Therefore, there is a risk of esophageal damage when RF energy is delivered anywhere in the LA, including particularly in the left atrium posterior wall. The nonuniform thickness of the posterior LA wall and the variable fibrofatty layer between the wall and the esophagus are risk factors that should be kept in mind during the ablation procedure. Esophageal arteries and the vagus nerve plexus on the anterior surface of the esophagus can also be affected by ablative procedures. Patients with AF and LA dilatation generally present a larger LA–esophageal contact area and thinner fat pads. Once esophageal necrosis develops, mediastinitis and fistula occur, connecting the esophageal lumen with the pericardium. It has also been reported that gastroesophageal reflux may facilitate mucosal injury, thus playing an additional role.^23^

Potential strategies to prevent esophageal injury include: (1) empirically reducing the power and duration of energy application on the posterior LA wall, (2) avoiding overlapping ablation lines, (3) monitoring esophageal temperature, (4) monitoring the esophageal position in relation to the posterior LA, and (5) educating the patient regarding signs and symptoms of esophageal injury.

Endoscopic evaluation postablation reveals a high (15%–48%) rate of incidence of esophageal erosion in patients undergoing RF for PV isolation.^24^ Predictors of esophageal injury and the potential for the development of esophageal fistula^24^ include persistent AF ablation, power of greater than 30 W, an increase in esophageal temperature to higher than 40°C, intraoperative pain (if sedation is used), and proximity of the esophagus to the LA wall.

#### Pericardial effusion and cardiac tamponade.

The occurrence of cardiac tamponade following AF ablation is rare,^25^ with an incidence rate of 0.98% per procedure and 1.46% per patient. It is the most frequent cause of death occurring in association with AF ablation.^8^ In particular, its incidence is higher when conducting AF ablation (0.8%–2.9%).^6,26,27^ This can be attributed to the complexity of the procedure, frequent manipulation with catheters, extensive ablation, and systemic anticoagulation.

Cardiac perforation leading to cardiac tamponade can occur (1) during transseptal puncture (puncture of the right atrial posterior wall or puncture the roof, appendage, or lateral LA wall); (2) during catheter manipulation (tear of the LA appendage or roof of the LA); or (3) during the delivery of RF energy (overheating with the development of steam pop, leading to myocardial rupture).

Clinically, cardiac tamponade presents as an abrupt fall in blood pressure. The use of intracardiac echocardiography (ICE) allows for the earlier detection of pericardial effusion^28^ and reduces the risk of cardiac tamponade.^29^

The majority of patients who develop cardiac tamponade can be managed by percutaneous drainage, but some may require surgical closure. The LA roof is susceptible to perforation that may not be responsive to pericardiocentesis. On the roof, the pericardium is not adherent to the LA.^28^

Steam pop due to tissue boiling related to the delivery of high levels of energy is a risk factor of cardiac tamponade. Limiting the energy delivered can significantly decrease the incidence of cardiac tamponade.^27^

#### Stroke and thromboembolic complications.

Thromboembolism is one of the most serious and feared complications of AF ablation. The incidence of periprocedural thromboembolic ranges from 1% to 7%.^7,8^ Silent cerebral ischemia detected on magnetic resonance imaging is substantial, with risk factors including the level of activated clotting time and cardioversion to sinus rhythm during the procedure.^30^

The risk of cerebral thromboembolism is at its highest in the first two weeks following ablation, with the most likely cause being the formation of char and/or thrombus at the sites of ablation.

#### Preprocedural management of anticoagulants.

The continuation of anticoagulation with warfarin reduces the risk of periprocedural stroke without increased bleeding complications.^31,32^ Di Biase et al. demonstrated that the combination of an open-irrigation ablation catheter and periprocedural therapeutic anticoagulation using warfarin may, in fact, reduce the risk of periprocedural stroke without increasing the risk of bleeding or pericardial effusion.

Novel oral anticoagulants (NOACs) can also be continued during the procedure. Di Biase et al. showed that an uninterrupted apixaban strategy is effective in preventing thromboembolic events without increasing the bleeding risk,^33^ while Cappato et al. suggested that the use of uninterrupted oral rivaroxaban is feasible, with a similar event rate to that of uninterrupted warfarin.^34^ Further, in a study by Calkins et al., anticoagulation with uninterrupted dabigatran was associated with less bleeding than that seen with uninterrupted warfarin.^35^

#### Phrenic nerve injury.

PN paralysis has been described after RF ablation and is a rare complication occurring in 0.48% of patients.^36^ Ablation near the right superior PV or within the superior vena cava can affect the right PN.^36^ Complete and partial recovery were observed in 66% and 17% of patients, respectively. Symptoms include dyspnea, hiccups, cough, and pain, while pleural effusion and atelectasis can also be seen. Fluoroscopy confirms the diagnosis.

To prevent PN injury, a number of strategies have been designed. These include ensuring antral PV ablation; high-output pacing to establish whether the PN can be captured from the proposed ablation site before ablation; PN mapping with anatomic tagging of its course using an electroanatomic mapping system; and monitoring of the diaphragmatic excursion with abdominal palpation, fluoroscopy, or intracardiac ultrasound while pacing the PN from the superior vena cava or subclavian vein during ablation.^37^

Direct monitoring of diaphragmatic compound motor action potentials (CMAPs) during ablation using diaphragmatic electromyography is another technique that has been suggested to further reduce the incidence of PN injury.^38,39^ CMAPs are recorded using body surface electrodes, esophageal electrodes, or a diagnostic catheter positioned in the hepatic vein. In some studies, a decrease in the amplitude of the myopotential by 30% has been reported to be more sensitive than abdominal palpation for predicting the subsequent reduction in diaphragmatic excursion and nerve injury.^40^

#### Vagal nerve plexus injury.

RF delivery to the posterior wall of the LA may cause injury to the vagal esophageal plexus, leading to pyloric spasm, gastric hypomotility, and a markedly prolonged gastric-emptying time.^41^ In the study by Shah et al., four of 367 patients who underwent AF ablation developed symptoms of vagal nerve injury within 48 hours after the procedure.^41^ Symptoms included abdominal bloating and discomfort hours to days after the procedure.^42,43^ Spontaneous recovery may require a time frame of up to one year. Prevention involves conducting lower-power and shorter-duration ablation on the posterior wall.

### Prevention and management of complications and collateral damage in epicardial ventricular tachycardia ablation

Ventricular tachycardia (VT) is a challenging arrhythmia for both patients and physicians alike to deal with. Antiarrhythmic medications have limited effectiveness and are often poorly tolerated.^44,45^ Meanwhile, catheter ablation is being adopted increasingly more frequently and appears to be reasonably effective.^46–48^ In 2012, a total of 2,927 VT ablation procedures were performed in the United States.^49^ Most VTs can be ablated endocardially but some require epicardial mapping and ablation.

#### Bleeding.

Hemopericardium is seen with pericardial access in about 8% of patients.^50^ Right ventricular puncture/laceration, coronary vessel puncture/laceration, and/or adhesion disruption are common reasons for hemopericardium. Double right-ventricular perforation could lead to extensive bleeding when the sheath is removed at the end of the case.

ICE plays an important role in identifying pericardial effusion, including being able to identify the wire if it is in the RV. Most cases of bleeding with RV punctures from the access needle are self-limited if the sheath is not advanced into the RV. Similarly, most small-vessel punctures or adhesion disruptions stop bleeding without intervention other than aspiration. However, the occurrence of major-vessel puncture or chamber laceration requires cardiac surgery to address.

#### Phrenic nerve.

The left PN should be identified by high output pacing, ranging from 20 to 50 mA with a 2-ms pulse width.^51^ When ablation needs to be performed adjacent to the PN, it can be displaced.^52^ Di Biase et al. demonstrated that controlled and progressive inflation of air and saline with careful monitoring of hemodynamic parameters appears to be the best strategy for preventing PN injury during epicardial ablation. A balloon catheter can also be used to separate the PN from the ablation catheter.^53^

As mentioned previously, monitoring of diaphragmatic excursion with abdominal palpation, fluoroscopy, or intracardiac ultrasound while pacing the PN from the superior vena cava or subclavian vein during ablation^37^ is another technique that can be used to prevent PN injury.

#### Epicardial coronary arteries.

Coronary arteries can be damaged by RF ablation.^54^ A coronary angiogram is routinely performed to define the relative location of the coronary arteries relative to the area of interest and establishing a distance of at least 5 mm away during this procedure from the coronary arteries is recommended. The adoption of multiple angiographic views should be completed to assess the distance appropriately.

### Prevention and management of complications and collateral damage in supraventricular tachycardia ablation

SVT ablation is performed to treat arrhythmias involving the upper chambers of the heart. This procedure aims to reduce symptoms of tachycardia and improve the patient’s quality of life. Once inside the heart, the catheters are used to stimulate and record electrical signals to induce the clinical arrhythmia, understand its mechanism of action, and identify the treatment site that would yield the best result for termination of the tachyarrhythmia. The success rate of an SVT ablation ranges between 93 and 97%^55,56^ and its associated complication rate is 0.8%.^5^

The major complications from this procedure are conduction system damage and cardiac perforation. In particular, inadvertent damage to the AV node or His bundle is the most feared complication during an SVT ablation. This phenomenon most commonly occurs in patients requiring slow-pathway modification for the treatment of atrioventricular nodal reentry tachycardia (AVNRT) because of the close proximity of the AV node to the anatomic area of the slow pathway. The slow pathway is usually located in the posteroinferior aspect of the triangle of Koch, while the AV node and His bundle are located in the anterosuperior aspect of this triangle. These anatomic landmarks can be blurred and not clearly well-demarcated in some patients, rendering slow-pathway modification particularly challenging. This occurs especially in young patients due to the vertical orientation of their hearts or elderly patients due to the posterior rotation of their hearts. The incidence rate of complete heart block in patients while attempting slow-pathway modification for treatment of AVNRT is 0.8%.^57^

More recent data have suggested an increased risk of late pacemaker implantation after AVNRT ablation. After AVNRT ablation, the risk of late pacemaker implantation was low but remained three times higher than that in patients without AVNRT and three times higher than the risk of periprocedural pacemaker implantation.^58^ Furthermore, ablation does not seem to be the cause of the heightened late pacemaker implantation risk.

There is a very minimal risk of inadvertent heart block in patients being treated by catheter ablation for typical atrial flutter because of the relatively far distance of the cavotricuspid isthmus to the AV node and His bundle. The same holds true for lateral wall accessory pathways. However, the risk is much higher for para-Hisian accessory pathways. The risk of inadvertent complete heart block while attempting a para-Hisian accessory pathway ablation is 3% to 5%.^59–61^

Cryoablation may carry a lower risk of AV blockage, but this mode of therapy is associated with a higher recurrence rate.^62–64^

## Conclusion

In summary, here, we reviewed the types of collateral damage associated with RF ablation of AF, VT, and SVT. We also discussed the types of collateral damage associated with each type of ablation procedure and the prevention measures available in each context.

It is very important to have a good understanding of the anatomy during RF ablation—not only what is in contact with the ablation catheter but also what is around the area being ablated. This can help both to enhance procedural outcomes and limit or avoid complications.
